# Ozonated saline intradermal injection: promising therapy for accelerated cutaneous wound healing in diabetic rats

**DOI:** 10.3389/fvets.2023.1283679

**Published:** 2023-11-06

**Authors:** Ahmed Hesham, Marwa Abass, Haanin Abdou, Reham Fahmy, Maha M. Rashad, Abdelnaser A. Abdallah, Wael Mossallem, Ibrahim F. Rehan, Asmaa Elnagar, František Zigo, Silvia Ondrašovičová, Ahmed F. Abouelnaga, Awad Rizk

**Affiliations:** ^1^Undergraduate Student, Faculty of Veterinary Medicine, Mansoura University, Mansoura, Egypt; ^2^Department of Surgery, Anesthesiology, and Radiology, Faculty of Veterinary Medicine, Mansoura University, Mansoura, Egypt; ^3^Veterinary Surgery, Oncology Centre, Mansoura University, Mansoura, Egypt; ^4^Biochemistry and Molecular Biology Department, Faculty of Veterinary Medicine, Cairo University, Giza, Egypt; ^5^Department of Internal Medicine and Infectious Disease, Veterinary Teaching Hospital, Faculty of Veterinary Medicine, Mansoura University, Mansoura, Egypt; ^6^Veterinary Clinical Supervisor, Al-Rahba Veterinary Clinic, Abu Dhabi, United Arab Emirates; ^7^Department of Husbandry and Development of Animal Wealth, Faculty of Veterinary Medicine, Menofia University, Shibin El Kom, Egypt; ^8^Department of Pathobiochemistry, Faculty of Pharmacy, Meijo University Yagotoyama, Nagoya, Japan; ^9^Department of Nutrition and Animal Husbandry, University of Veterinary Medicine, and Pharmacy, Košice, Slovakia; ^10^Department of Biology and Physiology, University of Veterinary Medicine, and Pharmacy, Košice, Slovakia; ^11^Department of Animal Behaviour and Management, Faculty of Veterinary Medicine, Mansoura University, Mansoura, Egypt

**Keywords:** ozone, wound healing, diabetes, histopathology, gene expression, rats

## Abstract

**Introduction:**

The use of ozonized water is gaining importance in medicine due to its effects on hyperglycemia and wound healing mechanisms.

**Methods:**

This experiment was conducted to assess the impacts of intradermal administration of ozonated water on acute skin wound healing in a diabetic rat model. Sixty-four adult male Wistar rats were randomly divided into two groups: an ozonated water group (O3W) and a control group (CG). Experimental diabetes was chemically induced in the rats by the intraperitoneal administration of 60 mg/kg streptozotocin. One week later, full-thickness skin surgical wounds (1 cm^2^) were created between the two shoulders of the rats under general anesthesia. The wounds were then daily irrigated with normal saline (CG) or intradermally injected with 1 mL of ozonated water at 10 mg/L O3W. Wound healing was evaluated through macroscopic analysis, measuring wound size, diameter, and percentage of contraction rate before wounding and at 3, 7, 9, 12, 14, 18, 21, 24, and 28 days post-wounding. On days 7, 14, 21, and 28 after induction of the wounds, the body weights and blood glucose levels of rats (8 per group) were measured before the rats were euthanized. Moreover, the morphological structure of the tissue, vascular endothelial and transforming growth factor (VEGF and TGF) affinity and gene expression were examined.

**Results:**

The O3W group had significantly lower blood glucose levels and wound size and gained body weight. Additionally, epithelial vascularization, stromal edema, TGF, and VEGF gene expression significantly improved in the O3W group.

**Discussion:**

Therefore, ozonated water has the potential to enhance and promote cutaneous wound healing in diabetic rats.

## 1. Introduction

The process of wound healing is complex and consists of several concurrent phases: coagulation, inflammation, proliferation, and remodeling (1). Upon injury, inflammatory cells are activated and secrete various growth factors, cytokines, and chemokines, which in turn stimulate resident cells (endothelial cells, fibroblasts, and keratinocytes) to migrate, proliferate, and form vascularized connective tissue, thus closing the wound (2).

Keratinocytes mainly mediate re-epithelialization of the wound, while dermal fibroblasts provide the matrix, and neovascularization is induced by endothelial cells (3). However, pro-inflammatory cytokines such as IL-1 and TNF-*α* can lead to chronic wounds or hypertrophic scars (4).

Diabetic wound healing is impaired by several factors, such as chronic inflammation, poor blood vessel formation, increased oxidative damage, and defective collagen production (5). High blood sugar levels in diabetes form advanced glycation end products (AGEs) that modify proteins and lipids in wound tissue (6, 7). AGEs disrupt the wound healing process either directly or through their receptors (7). They affect the movement of inflammatory cells across the blood vessel wall and prolong the inflammatory phase (8). Additionally, they impede the arrival of endothelial progenitor cells, which are necessary to grow new blood vessels. Furthermore, they alter the structure of fibroblast membranes, leading to early cell death and reduced collagen synthesis and deposition (7, 9). These events prevent diabetic wounds from shrinking and closing (10).

Diabetic wounds need to be protected from germs and infections using appropriate dressings that provide the right conditions for healing. The dressing should keep the wound moist, release bioactive substances continuously and effectively, and not break down too quickly during the healing process (11, 12).

As an adjunctive therapy for wound healing, ozone therapy offers a non-invasive, cost-effective, and safe method that enhances tissue repair through its strong oxidative action. Ozone can eliminate microorganisms without inducing resistance (13) and modulate the local inflammatory response by inducing the synthesis and secretion of cytokines and growth factors, such as platelet-derived growth factor (PDGF), vascular endothelial growth factor (VEGF), transforming growth factor (TGF), and epithelial growth factor (EGF) (14–16).

Ozone can be applied to skin wounds as a gas or dissolved in water or oil (ozonized solutions). Ozonized solutions have the advantages of avoiding ozone inhalation and creating a moist environment that favors the interaction of ozone with biomolecules. Ozonized water is convenient and easy to use in difficult areas, but it must be used immediately after ozonation because ozone quickly degrades in water. Oil, on the other hand, releases ozone molecules slowly into the tissue to stabilize the gas (14, 17).

However, there is a lack of a standardized protocol for treating skin wounds using these solutions, and no comparative studies have assessed their pros and cons (16). The goal of this study was to explore the possible benefits of ozonized water on skin wounds in diabetic rats, as ozonized solutions are believed to facilitate tissue repair.

## 2. Materials and methods

### 2.1. Ethical approval

**Figure 1:**
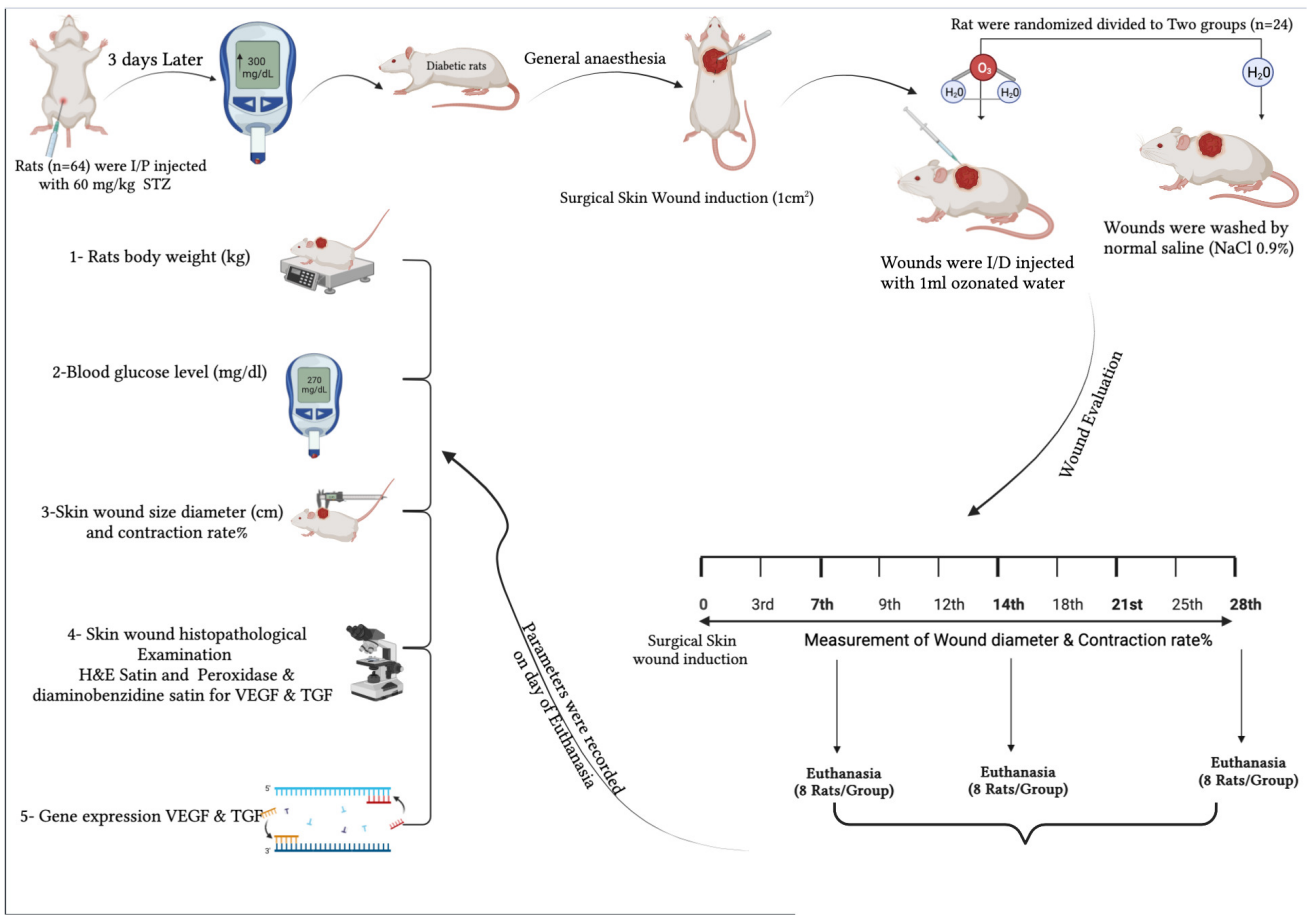
The schematic cartoon of the experimental strategy. It was designed by the authors of the manuscript (created with Biorender.com with permission).

All experiments were carried out in accordance with relevant guidelines and regulations. The Mansoura University Animal Care and Use Committee reviewed and approved this experiment, documented with code MU-ACUC (MV.R.22.11.25). All procedures were reported following the format specified by ARRIVE (18). The schematic cartoon of the experimental strategy is represented in (Figure 1).

### 2.2. Animals

This study used 64 adult-male Wistar rats, aged 12 weeks, weighing between 200 and 250 g. They were kept in cages with a light cycle of 14 h of light and 10 h of darkness in a temperature-regulated environment at 20°C. They had access to solid food before surgery, pasty food after surgery, and free water access. Male rats were selected to reduce variation as they did not influence the hormonal cycle of the cell response. Animals were randomly assigned to two experimental groups (*n* = 32). Weight was monitored throughout the study, and fasting blood glucose measurements were repeated on the day of euthanasia to confirm the diabetic state.

### 2.3. Induction of diabetes

Food was withdrawn from rats for 12 h prior to the induction of diabetes, after which they were intraperitoneally injected with freshly prepared streptozotocin (60 mg/kg body weight, STZ; Santa Cruz Biotechnology Inc., Heidelberg, Germany) to induce type I diabetes. STZ was thawed in citrate buffer (0.1 M and pH 4) immediately before use (19). To avoid acute hypoglycemia, the rats were administered a glucose solution (500 mL, Glucose 10%, Ultimate Pharma, Egypt) on the day following the STZ injection. Blood glucose levels were measured 72 h after the STZ injection using a Glucometer^®^ device [CareSens II, Pharmaco (NZ) Ltd., Auckland, New Zealand]. The rats showed glucose levels higher than 300–550 mg/dL and were monitored for one week for morbidity and mortality prior to the experiment. The weights of diabetic rats were measured throughout the study, and fasting blood glucose measurements were repeated on the day of euthanasia to confirm the diabetic state.

### 2.4. Ozonated water preparation

An ozone generator (Medical Ozone Generators of Medozons; Elsenfeld, Herrmann Apparatebau GmbH., Germany) at ambient temperature (20°C) and a flux rate of 1 L/min was used to produce ozonated water with a concentration of 10 mg/L. The cold, double-distilled water was injected into the storage tank of the medical ozone generator for 5 min. The generated ozonated water was used immediately (20–22).

### 2.5. Wound induction and experimental groups

The same experienced surgeon performed all the operations. The rats were subjected to general anesthesia by intraperitoneal administration of ketamine (60 mg/kg; Ketamax 5%, Troikaa, India) and medetomidine hydrochloride (1 mg/kg; Domitor 1‰, Pfizer, Portugal) and intramuscularly receiving meloxicam (0.3 mg/kg; 20 mg/mL; Metacam, Boehringer Ingelheim Co., Germany) before the surgical procedure. The dorsal region between the two scapulae was shaved and aseptically prepared. A full-thickness skin excision wound of 1 cm × 1 cm was created by removing 1 cm^2^ of skin from the prepared surgical area. Rats were intramuscularly injected with a single dose of meloxicam (0.3 mg/kg) for analgesic effects.

The first group was the CG (control group with a full-thickness skin defect), which consisted of 25 diabetic rats subjected only to injury, and the wound was irrigated with 0.9% sodium chloride solution (500 mL, sodium chloride 0.9%, Ultimate Pharma, Egypt). The second group was the O3W (a full-thickness skin defect treated with ozonated water group), which contained 25 diabetic rats that received an intradermal injection of 1 mL of ozonized water immediately after the skin wound was induced, and continued to receive daily injections for 28 days following the injury.

The rats were kept in individual cages at room temperature with free access to commercial feed and drinking water. All rats’ body weight (mg) and blood glucose level (mg/dL) were measured periodically at baseline and 0, 7, 14, 21, and 28 days post-surgery. Eight rats from each group were euthanized under general anesthesia by intraperitoneal injection of thiopental Na (100 mg/kg; 25 mg/mL; Adwia; Egypt) on days 7, 14, 21, and 28 post-injuries.

### 2.6. Macroscopic wound evaluation

A digital camera photographed wounds on the day they were made (Day 0) and then every 3rd, 7th, 9th, 12th, 14th, 18th, 21st, and 25th day until the 28th day post-wounding. A digital caliper was used to measure the wounds and calculate the size of wounds in two groups. The percentage of the wound contraction area was estimated using the following equation:


$$\mathrm{Wound}\;\mathrm{contraction}\%=1-\left(A_{d}/A_{0}\right)\times 100$$

where *A*_0_ is the wound area on day zero and *A*_d_ is the wound area on the related days to quantify the wound healing process and evaluate healing efficacy, as mentioned previously (23).

An expert observer blindly reported Visual observation of wounds is based on a modification of field (24) to detect the formation of wound scars, exudate painful reactions, and porphyria (red tears that accumulate on the medial canthus of the rat’s eye). The quantitative score was absent, mild, moderate, severe, or extremely severe.

### 2.7. Histological analysis and examination

Histological preparations were described previously (24). Briefly, cutaneous wound tissue samples were cut into 3–4 mm-thick slices, fixed with 10% neutral buffered formalin (NBF), dehydrated with increasing concentrations of ethanol solutions, cleared with xylene, and embedded in paraffin. Paraffin-embedded tissues were sectioned at 4–6 μm thickness using a microtome and stained with H&E to observe the general tissue morphology. A Leica microscope under different magnification powers (CH9435 Hee56rbrugg) (Leica Microsystems, Switzerland) was used for examining the H&E-stained slides.

Histological sections were used to evaluate the wound healing process by a qualified observer in a blind manner (25). In brief, it was a numerical scale that studied reepithelization, connective tissue formation, neovascularization, fibroblasts, and polymorphonuclear leucocytes (PMNL). The score ranged from zero to three (“0” = absence of a parameter, “1” = mild exposure of a parameter, “2” = moderate exposure of a parameter, and “3” = extensive exposure of a parameter). The total mean value was calculated and used for statistical analysis.

### 2.8. Immunohistochemistry staining protocol

The avidin-biotin-peroxidase complex (ABC) method was used to perform immunohistochemistry on paraffin tissue sections that had been fixed to slides with a positive charge. Rabbit anti-VEGF receptor-2 (Elabscience Cat# E-AB-63481, Dil.: 1:50) and rabbit anti-TGF beta-1 (Arigobio, Cat# ARG56429, Dil.:1:50) polyclonal antibodies were used. Sections from each group were incubated with the previously described antibodies. Subsequently, the chemicals involved in the ABC technique (Vectastain ABC-HRP kit, Vector Laboratories) were used. Marker expression was identified using peroxidase and stained with diaminobenzidine (DAB, produced by Sigma) to distinguish the antigen-antibody complex.

Negative controls were integrated with non-immune serum instead of primary or secondary antibodies. Immunostained sections were examined and photographed using a Leica microscope at various magnifications (CH9435 Hee56rbrugg; Leica Microsystems, Switzerland).

### 2.9. Quantitative scoring of immunohistochemical results “area percentage”

The area percentage of VEGF and TGFβ immunoreactivity was quantified using the Leica QWin500 image analysis system (England) in six high-power fields (400×) with positive brown staining in each serial section of the groups under investigation. The system comprised a Leica microscope, a color video camera, a color monitor, and a Leica IBM PC hard disk connected to the microscope, all operated by the Leica QWin500 software. The mean and standard deviation (mean ± SD) of the percentage area for each antibody were calculated and reported statistically.

### 2.10. Gene expression

The relative mRNA expression of VEGF and TGF*β*1 in the skin was measured by qRT-PCR, using GAPDH as an internal control (26). Total RNA was extracted from approximately 50 mg of skin tissue using a total RNA extraction kit (Vivantis, Malaysia), and its quality and quantity were verified. cDNA was synthesized using M-MuLV reverse transcriptase (NEB#M0253) and performed using real-time PCR by SYBR Green dye to quantify the amplification of each target gene. The primer sequences for VEGF were 5′-GCAATGATGAAGCCCTGGAG-3′ (forward) and 5′-GCTTGTCACATACGCTCCAG-3′ (reverse); TGF-*β*1, 5′-CACTCCCGTGGCTTCTAGTG-3′ (forward) and 5′-GGACTGGCGAGCCTTAGTTT-3′ (reverse) (27). The thermal cycling conditions were 95°C for 5 min (initial denaturation), followed by 40 cycles of 95°C for 15 s, 60°C for 30 s, and 72°C for 30 s. Negative controls without templates were included (28). Each qRT-PCR was performed in triplicate on three biological samples (29). The comparative 2^−ΔΔCT^ method was used to calculate relative expression levels (30).

### 2.11. Statistical analysis

The collected data were statistically analyzed using SPSS software (SPSS Statistics 16). The Kolmogorov–Smirnov test was used to test the normality distribution of the data. Normally of the distributed numerical data were presented as mean and standard deviation, and repeated measures outcomes were analyzed by repeated measures ANOVA for differences over time and between groups. All data were considered statistically significant if the *p*-value was ≤0.05.

## 3. Results

### 3.1. Body weights and blood glucose levels of rats

**Figure 2:**
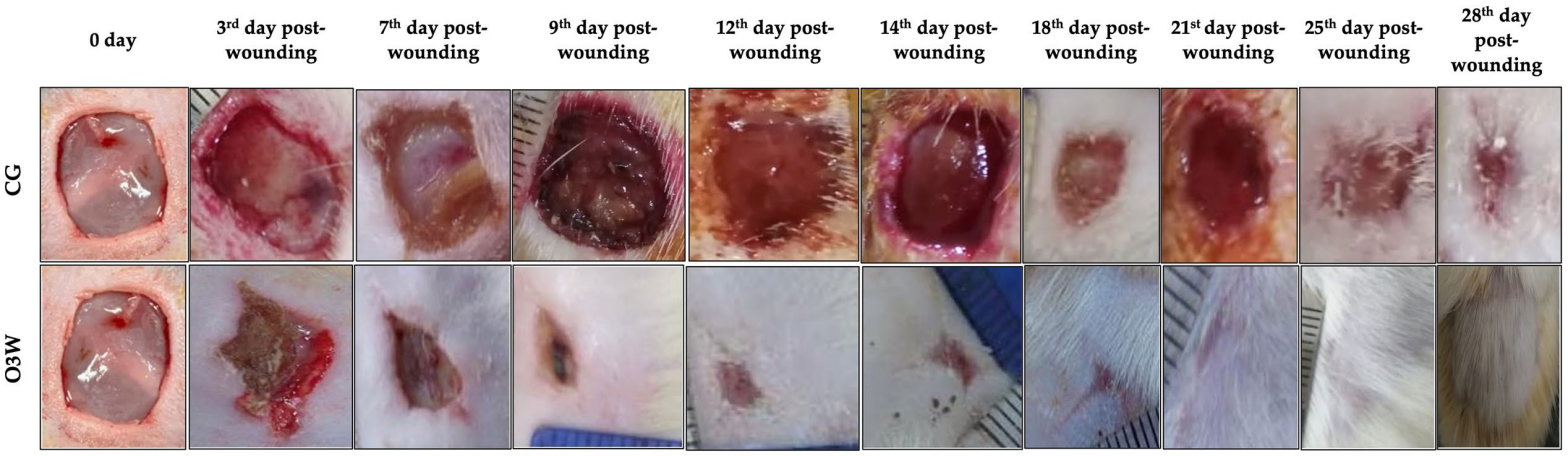
Level of **(A)** body weight (mg) and **(B)** blood glucose level (mg/dL) in rats before induction of diabetes mellitus in rats and after diabetic induction at 0, 7th, 14th, 21st, 28th day post- wounding (mean ± SD).

One week after the injection of STZ, the mean average blood glucose levels were increasing, and the rats had lost body weight without a significant difference (*p* > 0.05) between groups. On days 7, 14, 21, and 28 post-wounding, the rats’ body weights had statistically declined in the CG compared to the O3W group (*p* ≤ 0.05) and baseline value. Furthermore, the blood glucose levels in the O3W group were significantly lower (*p* ≤ 0.05) than the CG at each measured time point post-operation (Figure 2).

### 3.2. Macroscopic assessment of the wound and wound kinetics and calculation

The open wound area decreased in both groups on days 3, 7, 12, 14, 18, 21, 25, and 28 post-wounding without excessive redness, irritation, or infection. In contrast to the CG, scar tissue appeared on the third day after the operation in the O3W group. Only the CG wounds had a clear, straw- or amber-colored exudate that was noticed in the first 7 days post-operation. Painful reactions at wound sites were noticed in the CG group until 25 days post-operation, while in the O3W group, painful reactions at wound sites only appeared on the 7th day post-wounding. Porphyria was recorded in the CG for 14 days post-operation (Table 1).

**Table 1:** Visual macroscopical assessment of wounds in a control (CG) and an ozonated water (O3W) group during skin wound healing in rats at 0, 3rd, 7th, 9th, 12th, 14th, 21st, 25th, and 28th days post-wound induction.

Post-wounding (days)	Scare formation		Exudate		Pain reaction		Porphyria	
	CG	O3W	CG	O3W	CG	O3W	CG	O3W
3rd	+	−	−	+	++++	++	++	−
7th	++	−	−	+	+++	++	++	−
9th	+++	−	−	−	+++	+	++	−
12th	+++	−	−	−	+++	−	+	−
14th	+++	−	−	−	++	−	+	−
18th	+++	+	−	−	++	−	−	−
21st	++++	++	−	−	+	−	−	−
25th	++++	++	−	−	+	−	−	−
28th	++++	++	−	−	−	−	−	−

“−”absence; “+” mild; “++” moderate; “+++” severe; “++++” extremely severe.

**Figure 3:**
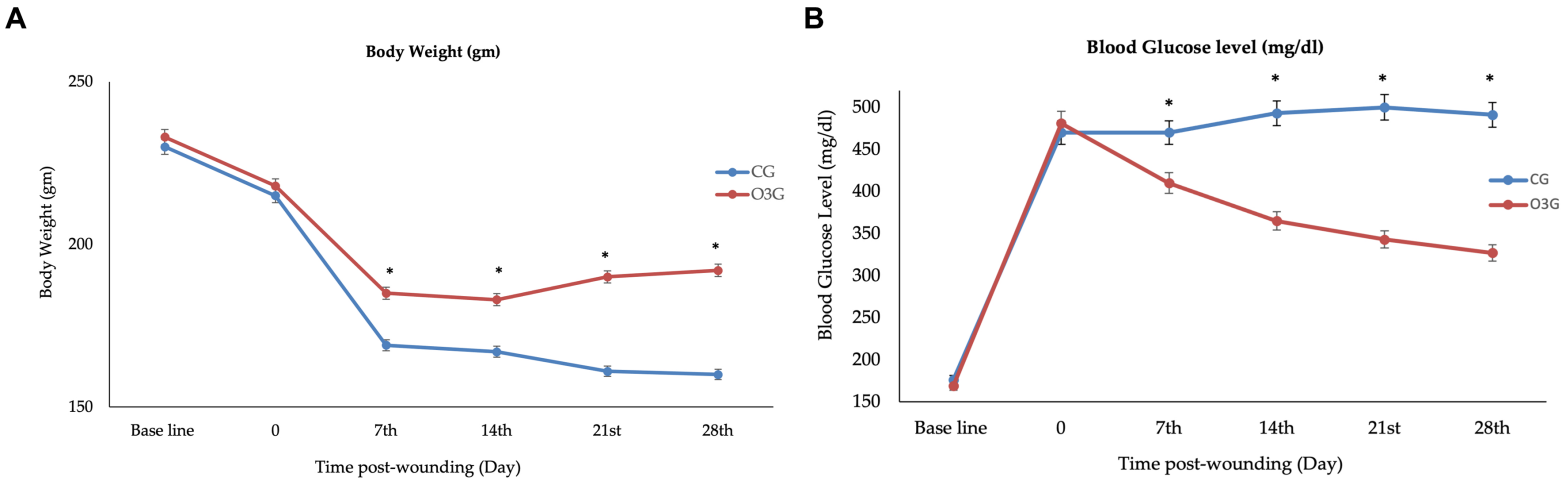
Photographs of **(A)** body weight (gm) and **(B)** blood glucose level (mg/ml) in a control (CG) and an ozonated water (O3W) group during skin wound healing in rats at 0, 7th, 14th, 21st, and 28th days postoperatively.

Moreover, the wound area was smaller and contracted rapidly in the O3W group compared to the CG at each measured time point. On average, the wounds were closed grossly in the O3W group (25 ± 2) days post-wounding. The degree of wound healing through the measured times is shown in (Figure 3).

**Figure 4:**
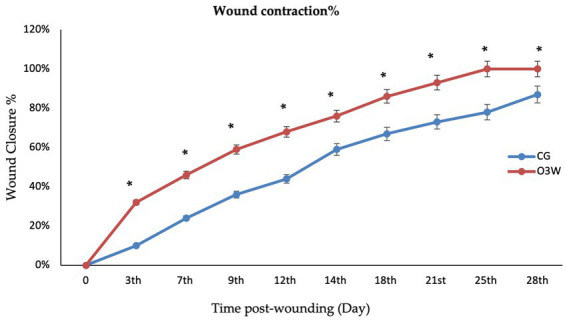
The wound closure rate % in the control (CG) and ozonated water (O3W) groups during skin wound healing in rats at 0, 3rd, 6th, 9th, 12th,15th, 21st, 25th, and 28th days post-wound induction.

There was a significantly higher percentage of wound area contraction within the same group than at baseline (*p* ≤ 0.05). However, O3W showed a significant decrease in wound area compared to the baseline and CG at the same time points on the 3rd, 7th, 9th, 12th, 14th, 21st, 25th, and 28th days post-wound induction (Figure 4).

The diameter of the wound area (cm^2^) in the O3W group significantly decreased at different measured time points (*p* ≤ 0.05). Additionally, the size of the O3W wound area was significantly lower (*p* ≤ 0.05; Table 2) than that of the CG at the 3rd, 7th, 9th, 12th, 14th, 21st, 25th, and 28th days post-wound induction.

**Table 2:** Measurements of the wound size diameter (cm^2^) in a control (CG) and an ozonated water (O3W) group during skin wound healing in rats at 0, 3rd, 7th, 9th, 12th, 14th, 21st, 25th, and 28th days post-wound induction (mean ± SD).

Time post-wounding (day)	CG	O3W
0 (day of surgery)	1.1 ± 0.7	1.1 ± 0.7
3rd	0.91 ± 0.09	0.76 ± 0.05^*,†^
7th	0.76 ± 0.007	0.56 ± 0.04^*,†^
9th	0.63 ± 0.3	0.45 ± 0.03^*^
12th	0.52 ± 0.04	0.16 ± 0.03^*,†^
14th	0.43 ± 0.07	0.1 ± 0.02^*^
18th	0.36 ± 0.07	0.02 ± 0.01^*,†^
21st	0.25 ± 0.06	0.001 ± 0.01^*,†^
25th	0.2 ± 0.04	0.0 ± 0.0^*,†^
28th	0.18 ± 0.04	0.0 ± 0.0^*^

^(*)^Means different between the same raw (between groups) are statistically significant at (*p* ≤ 0.05), while ^(†)^means different in the same column (within the group) are statistically significant at (*p* ≤ 0.05) for different time point.

### 3.3. Histological analysis and examination

**Figure 5:**
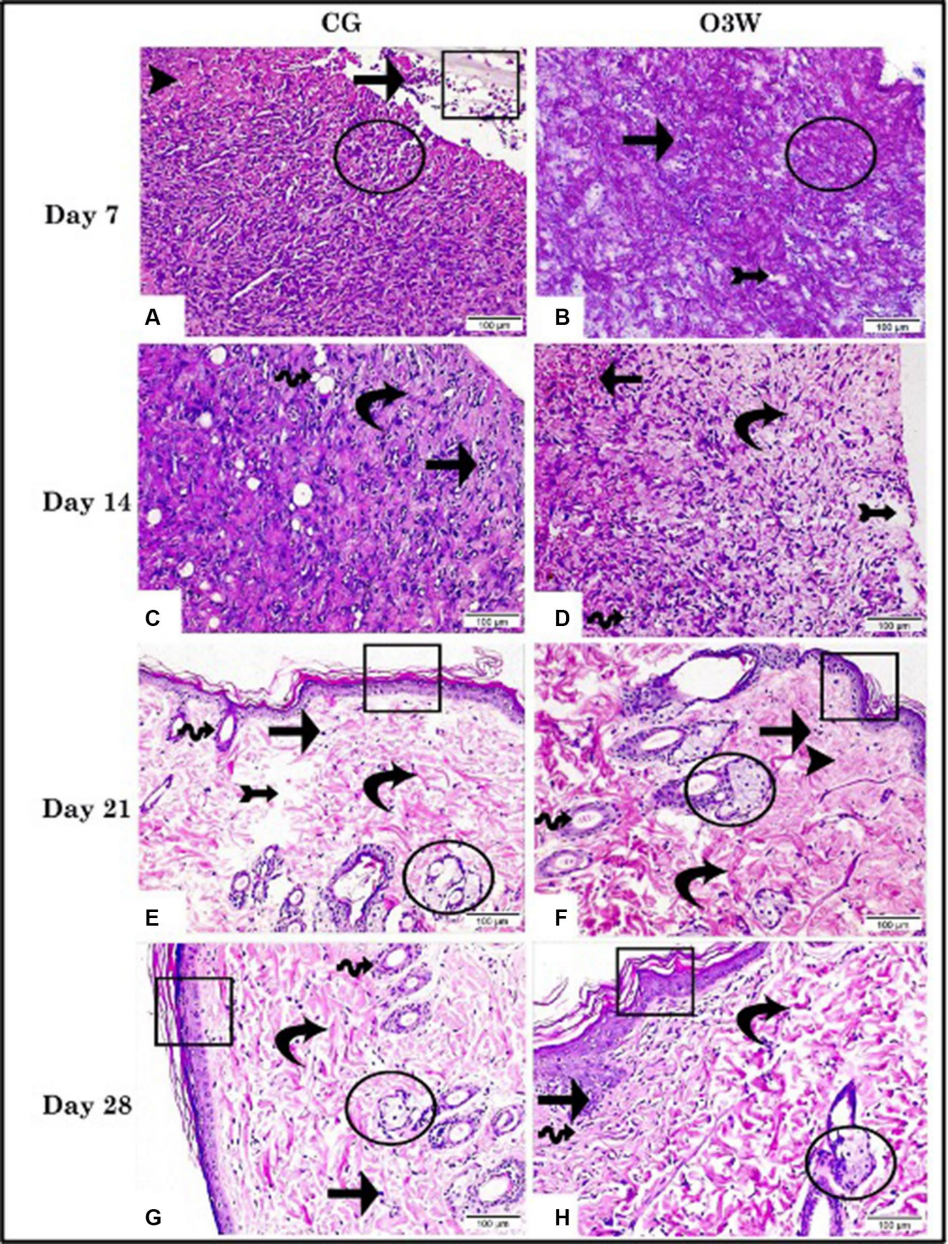
Photomicrographs demonstrated the histopathological alterations in skin wound tissue sections between a control (CG) represented in **(A,C,E,G)** and an ozonated water (O3W) represented in **(B,D,F,G)** group (hematoxylin & eosin stain, 200× & scale bar = 100 μm) at the 7th, 14th, 21st, and 28th days post-wounding. Infiltration of mononuclear inflammatory cells (arrow); granulation tissue (arrow head); free RBCs, fibrin threads, and epidermal layer (rectangle); bands of collagen fibers (circle); newly formed blood vessels (wave arrow); thick bands of collagen (curvy arrow); interstitial edema (arrow with tail); newly formed skin glands (circle).

Hematoxylin and eosin staining of a skin wound section on the 7th day post-wounding in the CG revealed an aggregation of dead cells of different origins at the wound surface suspended between thick bands of collagen fibers (Figure 5A). Additionally, infiltration of mononuclear inflammatory cells, free red blood cells, fibrin threads, and necrotic areas was observed. In the O3W group, dead and inflammatory cells were collected between the thick bands of collagen fibers and fibrin threads on the wound surface. Areas of interstitial edema led to the dispersion of fibrous connective tissue (Figure 5B).

On the fourteenth day post-wounding, the CG showed well-organized granulation tissue assembled with thick collagen bands, evidenced inflammatory cell infiltration, and newly formed blood vessels (Figure 5C). The O3W group exhibited well-organized granulation tissue with intact architecture encircling small, newly formed blood vessels. Some areas were marked by interstitial hemorrhage, mononuclear inflammatory cells, and interstitial edema (Figure 5D).

At 21 days post-wounding, CG revealed the formation of a thin epidermal layer, migrating epidermal cells, a thick bundle of collagen, a few mononuclear inflammatory cells, and interstitial edema, leading to dispersion between fibrous connective tissue and newly formed skin glands (Figure 5E). The O3W group developed a thin epidermal layer, well-organized granulation tissue, few inflammatory cells, thick collagen bundles, high numbers of migrating epidermal cells, and newly formed skin glands (Figure 5F). At 28 days post-wounding, CG showed a thin epidermal layer, migrating epidermal cells, a thick collagen bundle, a few mononuclear inflammatory cells, and a few newly developed skin glands (Figure 5G). Simultaneously, the O3W group showed a thick epidermal layer, few inflammatory cells, and regular dermis assembly in a distinct region; however, parallel-organized collagen fibers were detected in other areas (Figure 5H).

The histological parameters were assessed: re-epithelialization, neovascularization, fibroblast proliferation, and granulation tissue formation. These parameters were statistically higher (*p* ≤ 0.05) in the O3W group than in the CG. The histological parameters registered an average score of 2 versus 1, 2.6 versus 1.5, 2.9 versus 2, and 3 versus 2.5 in the O3W group compared to the CG at the 7th, 14th, 21st, and 28th days post-wounding. In contrast, the mononuclear cell infiltration and edema significantly declined (*p* ≤ 0.05) in the O3W group compared to the CG, with average scores of 1.3 versus 1.9, 0.9 versus 1.5, 0.6 versus 1, and 0.49 versus 1, respectively, at different time points post-wound induction (Table 3).

**Table 3:** Histological score of wound healing process (mean ± SD) by hematoxylin and eosin (H&E) staining in a control (CG) and an ozonated water (O3W) group at 7th, 14th, 21st, and 28th days post-wounding.

Day post-wounding	Groups	Formation of epithelium	Formation of new vessels	Formation of granulation tissue	Proliferation of fibroblasts	Infiltration of inflammatory cells
7th	CG	1 ± 0.9	0.43 ± 0.61	1.62 ± 0.15	0.91 ± 0.57	1.87 ± 0.71
	O3W	2.03 ± 0.1^*^	2.03 ± 0.80^*^	2.36 ± 0.53^*^	2.08 ± 0.41^*^	1.37 ± 0.31^*^
14th	CG	1.87 ± 0.47	0.90 ± 0.11	1.89 ± 0.33	1.91 ± 0.07	1.49 ± 0.60
	O3W	2.71 ± 0.07^*^	2.58 ± 0.42^*^	2.59 ± 0.39^*^	2.53 ± 0.25^*^	0.97 ± 0.32^*^
21st	CG	2.04 ± 0.08	1.69 ± 0.02	2.06 ± 0.60	1.80 ± 0.19	1.09 ± 0.21
	O3W	3.00 ± 0.00^*^	2.83 ± 0.17^*^	2.9 ± 0.08^*^	2.97 ± 0.01^*^	0.67 ± 0.11^*^
28th	CG	2.52 ± 0.47	2.02 ± 0.52	2.29 ± 0.41	2.83 ± 0.07	0.97 ± 0.56
	O3W	3.00 ± 0.00^*^	3.00 ± 0.00^*^	3.00 ± 0.00^*^	3.00 ± 0.00^*^	0.47 ± 0.21^*^

^(*)^Means different between the same raw (between groups) are statistically significant at (*p* ≤ 0.05).

### 3.4. Immunohistochemistry staining evaluation

**Figure 6:**
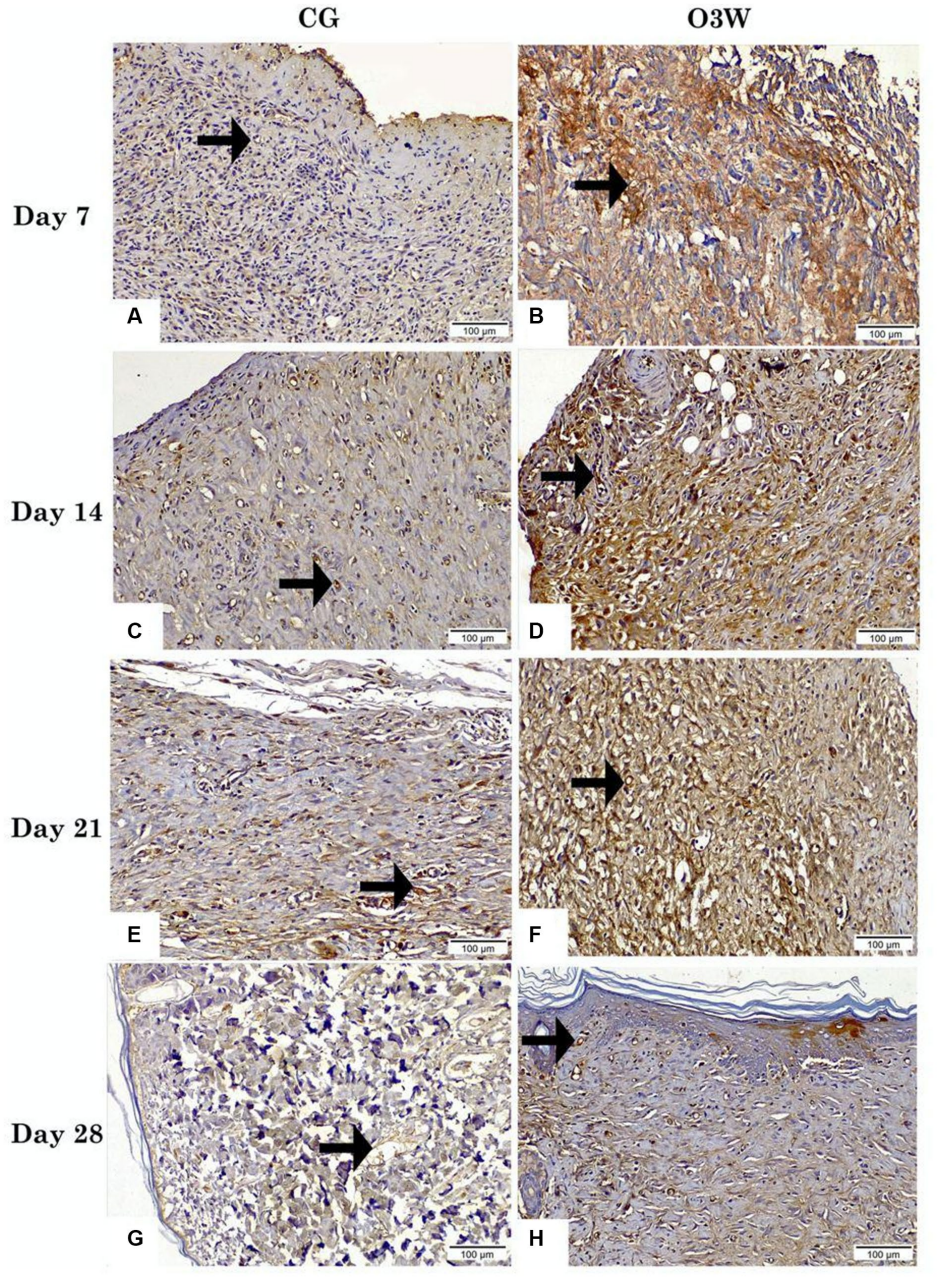
Photomicrographs displayed the reactivity of VEGF in skin tissue sections between a control (CG) represented in **(A,C,E,G)** and an ozonated water (O3W) represented in **(B,D,F,G)** group (VEGF antibody, magnification power = 200× & scale bar = 100 μm) at days 7th, 14th, 21st and 28th post-wounding. Arrows are indicated by blood vessels.

Immunohistochemical staining of skin wounds at different time points post-wounding in the CG displayed scarce positive expression of VEGF as membrane reactivity along blood vessels, with no significant difference (*p* ≥ 0.05) between all control groups at the measured time points (Figures 6A,C,E,G).

In contrast, the O3W group at days 7, 14, 21, and 28 post-wounding showed the highest, moderate, and low positive VEGF membranous and nuclear reactivity along blood vessels, respectively, with a significant difference (*p* ≤ 0.05) from all the CG. VEGF expression differed significantly (*p* ≤ 0.05) within the same O3W group at different time points (Figures 6B,D,F,H).

**Figure 7:**
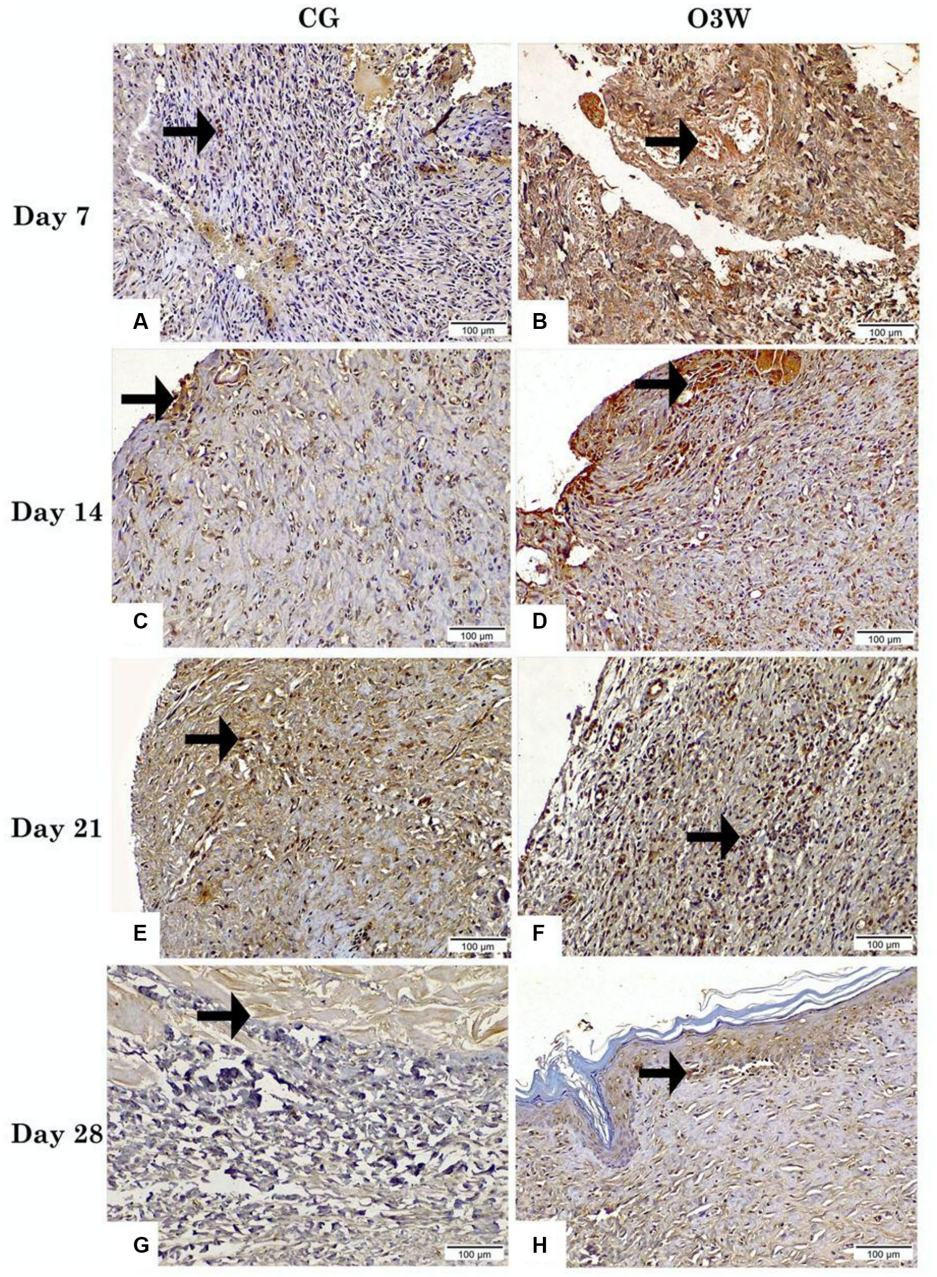
Photomicrographs presented the expression of TGFβ in skin tissue sections between a control (CG) represented in **(A,C,E,G)** and an ozonated water (O3W) represented in **(B,D,F,G)** group (TGFβ_Antibody, magnification power = 200× & scale bar = 100 μm) on days 7th, 14th, 21st, and 28th post-wounding. Arrows indicate TGF*β* nuclear reactivity.

In the CG, days 7, 14, 21, and 28 showed the lowest positive expression of TGF*β* concerning nuclear reactivity (Figures 7A,C,E,G). The expression of TGF*β* in the CG on the 7th day post-wounding revealed no significant difference (*p* ≥ 0.05) compared to that in the CG on the 14th and 21st days post-wounding. However, the CG on day 7 displayed a statistically significant decrease (*p* ≤ 0.05) compared to the CG on day 28. On the other hand, TGF*β* expression in the CG showed no significant difference (*p* ≥ 0.05) between the groups on the 14th, 21st, and 28th days after the induction of wounds.

TGF*β* expression in O3W on the 7th-day post-wounding demonstrated the highest positive TGFβ nuclear reactivity, with a significant difference (*p* ≤ 0.05) compared to the CG. Moreover, on the 14th day post-wounding, the CG and the O3W group exhibited high positive TGFβ nuclear reactivity, with a significant difference (*p* ≤ 0.05) from the CG and the O3W on the 7th day post-wounding. On the 21st day post-wounding, there was a moderately positive TGFβ nuclear reactivity (*p* ≤ 0.05) and a significant difference (*p* ≤ 0.05) between the CG and the O3W group on the 7th and 14th days post-wounding.

At the 28th day post-wounding, few positive TGF*β* nuclear reactivities were observed, with a significant difference (*p* ≤ 0.05) between the CG at the 21st and 28th days post-wounding and between O3W at the 7th, 14th, and 21st days post-wounding. However, the difference was not significant (*p* ≥ 0.05) with the CG on days 7 and 14 (Figures 7B,D,F,H).

### 3.5. Quantitative scoring of immunohistochemical staining “area percentage”

**Figure 8:**
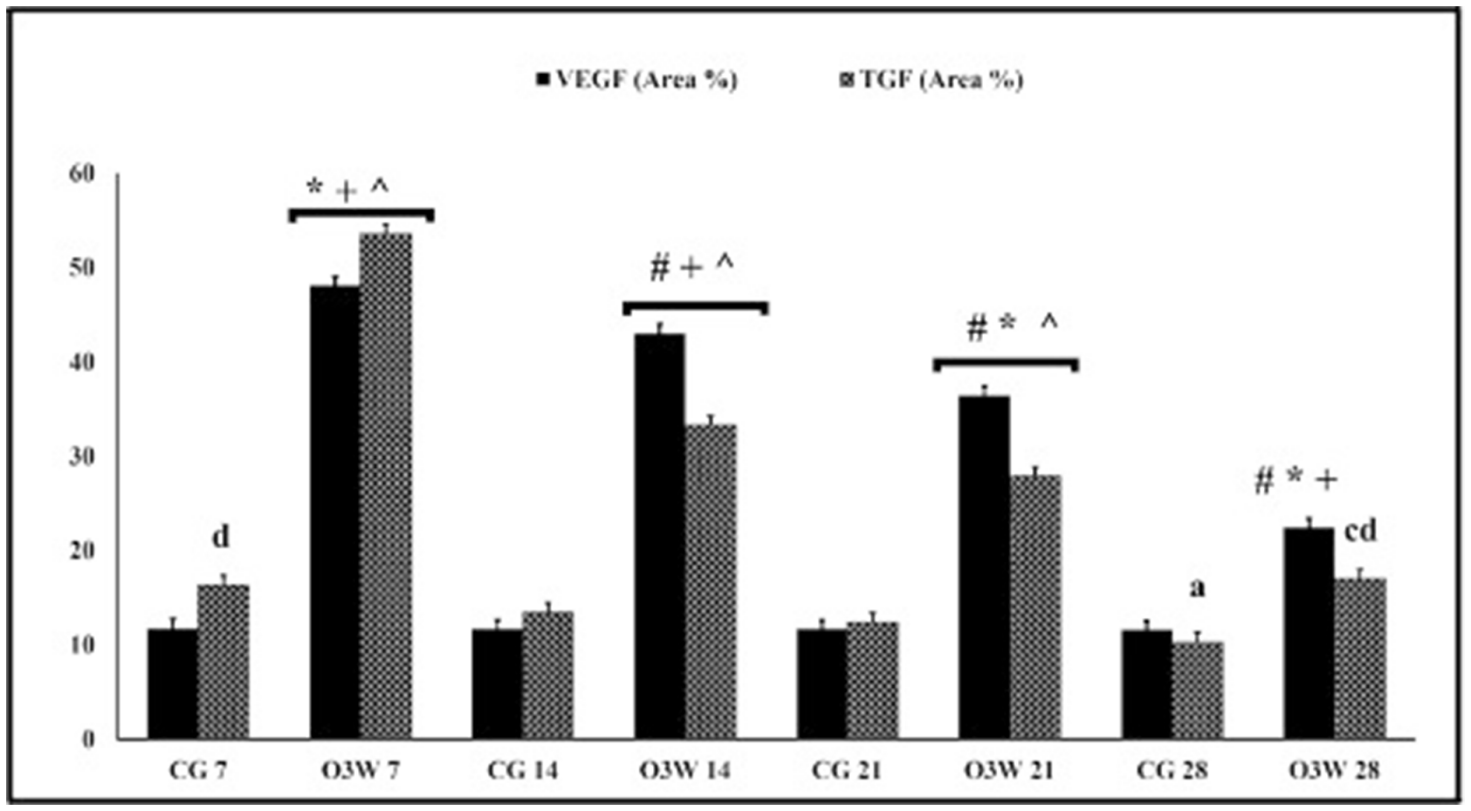
The immune scoring area % of VEGF and TGF*β* in skin tissue sections between a CG and an O3W group at 7th, 14th, 21st, and 28th post-wounding. Values expressed as mean ± SD (area %), ^#^significant vs. negative O3W 7, ^*^significant vs. O3W 14, ^+^significant vs. O3W 21, ^^^significant vs. O3W 28. ^a^significant vs. CG 7, ^c^significant vs. CG 21, ^d^significant vs. CG 28. Different superscript (^+,#,^,*,a,c,d^) indicate statistically significant differences at *p* ≤ 0.001.

The immune scoring area percentage of both anti-VEGF and anti-TGF*β* antibodies in skin tissue sections displayed significantly more intense staining (*p* ≤ 0.001) in the O3W group than the CG at 7th, 14th, 21st, and 28th post-wounding. Moreover, the qualitative immunohistochemical analysis of VEGF and TGF*β* was substantially enhanced and significantly improved (*p* ≤ 0.001) in the O3W group at different time points (Figure 8; Table 4).

**Table 4:** Immune scoring area% of vascular endothelial growth factor (VEGF) and transforming growth factor (TGF) in a control (CG) and an ozonated water (O3W) group during cutaneous wound healing in diabetic rats at 7th, 14th, 21st, and 28th days after induction of wounds.

Time post-treatment (day)	Group	VEGF (area %)	TGF (area %)
7th	CG	11.7 ± 0.04	16.3 ± 0.38
	O3W	48 ± 0.61^*,†^	53.5 ± 0.63^*,†^
14th	CG	11.6 ± 0.59	13.4 ± 0.55
	O3W	42.9 ± 0.95^*,†^	33.2 ± 0.78^*,†^
21st	CG	11.6 ± 0.57	12.3 ± 0.9
	O3W	36.4 ± 0.46^*,†^	27.8 ± 0.97^*,†^
28th	CG	11.5 ± 0.61	10.3 ± 0.01
	O3W	22.4 ± 0.09^*,†^	17 ± 0.04^*,†^

^(*)^Means differences between the same raw (between groups) are statistically significant at (*p* ≤ 0.05), while ^(†)^means differences in the same column (within the group) are statistically significant at (*p* ≤ 0.05) for different time points.

### 3.6. Relative expression of VEGF and TGF β1 mRNA genes

**Figure 9:**
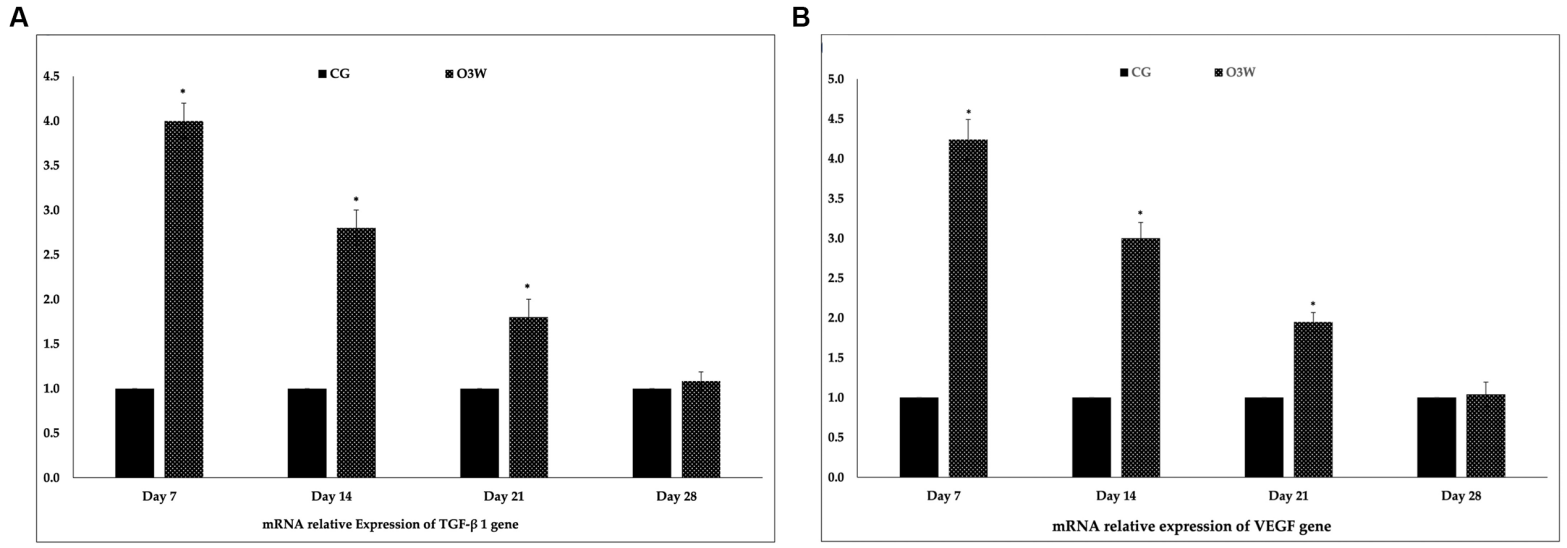
Mean and standard deviation of relative expression affinity genes between control and O3W groups at 7th, 14th, 21st, and 28th days post-wound induction in diabetic rats: **(A)** VEGF mRNA gene **(B)** TGF*β*1 mRNA gene.

The VEGF and TGF*β*1 mRNA levels showed a significant upregulation (*p* ≤ 0.05) in the O3W groups compared to the CG group on the 7th, 14th, and 21st days post-wounding. However, no significant increase was observed in the levels of both the VEGF and TGF*β* mRNA genes on the 28th day after the wound induction (Figure 9).

## 4. Discussion

Diabetes mellitus, a chronic hyperglycemic disorder, leads to impaired wound healing, ulcers, gangrene, and amputation of the affected region (31). Consequently, there is a global effort to develop drugs that can accelerate wound closure and prevent infection. To design such molecules, an animal wound model that mimics the conditions and molecular changes of a chronic wound in a diabetic patient is required (32).

Recently, ozone treatment has become an established therapy in many countries for clinical practice; however, its use remains limited (33, 34). Ozone therapy has been shown to enhance wound healing and regulate the immune system (33) due to its antibacterial and antifungal properties (17, 35), its effects on the production of pro-inflammatory cytokines such as TNF-*α* and IL-1, and its effects on adaptive inflammatory responses (36, 37), as well as keratinocyte growth and differentiation (36, 38). This review aimed to determine the efficacy of O3W as an advanced therapy for treating full-thickness wounds in diabetic rats.

The effects of ozone therapy on the healing of various tissues vary depending on the protocol used (35, 39–41). Previous studies have investigated the effects of ozone gas on wound healing in non-diabetic humans, rats, dogs, and cats (42, 43). In contrast, a study (44) measured the effects of ozonated olive oil and sunflower ozonized oil in a topical form of O3 in treating acute cutaneous wound injuries in non-diabetic rats and guinea pigs. However, the gas or oil forms of ozone require more time, and the procedure must be well-defined regarding gas flow, O3 concentration, oil volume, and temperature (45).

Previous investigations (45–47) approved that there were no significant differences between two aqueous and oily forms of ozone therapy, and both forms had improved cutaneous wound healing in horses. So that both forms of ozone can be used for wound treatment in horses. Furthermore, a few studies (48, 49) have mentioned that aqueous ozone results in less oxidative stress than gaseous ozone and is less cytotoxic, which is evidence of the safety of aqueous ozone. For these reasons, the current research uses ozonized water for cutaneous wound treatment in diabetic rats.

An ozone concentration of 0.1 mg/L yields a low impact on oral cavity bacterial control (49), but a high concentration of 5–8 mg/L yields good results (48). This is consistent with the current study, which found that 10 mg/L was superior to 3 mg/L for rapid skin wound closure in diabetic rats (pilot study unpublished data), as well as in the previous studies (20, 22) used the high concentration in orthopedic surgeries.

Administration of intravascular streptozotocin and injection of streptozotocin monohydrate can cause a reduction in insulin levels and lead to hyperglycemia within a short period, as reported in previous studies (50). Streptozotocin (STZ) is a natural substance that kills cells, particularly those in the pancreas that make insulin. When streptozotocin is injected, it destroys beta cells in the islets of Langerhans. To evaluate the sublethal concentrations, glucose levels were increased in rats exposed to potassium dichromate blood. The analysis of blood parameters has become increasingly significant in recent years due to their relevance in diagnosing various abnormalities (51, 52). STZ has severe harmful effects on various body organs, and it is possible that STZ toxicity, rather than DM complications, causes mortality in rats (53).

Similar to our results, the previous study (12) recorded that about 20% of the rats’ body weight was lost within a week after the injection of STZ. This was a result of excessive dehydration and a lowering of insulin levels post-injection of STZ, consequently inhibiting glucose uptake by various body cells and leading to the loss of fat and muscle. In albino rats, red tears that accumulated in the median canthus of the eye were considered a sign of a lack of grooming and pain (54). This experiment reported porphyrin in the CG on the 7th day post-injury.

Using a single dose of meloxicam to reduce postoperative pain was based on previous research (55), which examined the impact of biological and chemical substances on wound healing in rats. Furthermore (56), concluded that anti-inflammatory drugs interfere with the assessment of wound healing (57, 58). demonstrated that NSAIDs can cause hypoglycemia. Moreover, it was reported that ozone therapy effectively reduces inflammation by reducing pro-inflammatory cytokines and activating the IL-10 anti-inflammatory cytokine (59).

The current investigation revealed a non-significant gradual decline in the body weights of rats and an elevation in blood sugar levels after the induction of diabetes and on the day of surgery (baseline value). However, both parameters improved post-wound induction at each measurement point in the O3W group compared to the CG, which may be explained previously (60), who reported that ozone reduced STZ-induced hyperglycemia and increased endogenous antioxidant defense levels in the pancreas.

The results showed that the wound size and contraction rate significantly decreased in the O3W group compared to the CG group at each time point post-wounding in the diabetic rat model. These results indicated that O3W improved acute skin wound healing. On day 7 post-wounding, the scar tissue-covered wound was without exudate, and the wound size and wound closure rate percentage in the O3W group were significantly decreased. A previous study suggested that topical exposure to O3W may affect granulation tissue formation during the wound-healing process more than the early formation of blood clots and inflammatory cells during the inflammation phase (61). Moreover, this finding agrees with the results reported for the effects of ozonated compounds in a guinea pig model (44).

The current experiment observed a notable decrease in the dimensions of injury in the group treated with ozone water (O3W) relative to the control group (CG), which could be attributed to its influence on the production of growth factors, stimulation of the antioxidant system, and induction of superoxide dismutase (62). Similar outcomes were also documented in (41).

The histological analysis revealed that the wounds treated with O3W had granulation tissue with reduced inflammatory cell infiltration and increased spindle-shaped cells, indicative of myofibroblasts. Moreover, the O3W group exhibited significantly enhanced vascularization and collagen synthesis compared to the control group. These findings indicated a more advanced and controlled healing process in the experimental group than in the control group. These results were also reported previously (63), who assessed the effects of ozone on accelerating wound closure in non-diabetic rats.

Fibroblasts are involved in various wound healing processes, such as re-epithelialization, collagen production, extracellular matrix restoration, wound remodeling, and the secretion of endogenous growth factors like PDGF, VEGF, FGF, and TGF*β* (64). In the current study, we observed enhanced TGF expression in the O3W group after wounding, consistent with the increased collagen deposition and fibroblast proliferation at the same time. Likewise, it was reported that these results indicated that O3 might stimulate the expression of TGF from both epidermal keratinocytes and dermal fibroblasts at the injured site (65).

An earlier investigation reported that VEGF expression in the epidermal keratinocytes increased gradually from day 1 to day 7 of the normal healing process of the wound (66). This agrees with the higher expression of VEGF observed in the O3W group after wounding. Moreover, H&E staining revealed enhanced vascularity in the O3W group. These effects may be related to hydrogen peroxide (H_2_O_2_) production by ozonation, which can directly or indirectly stimulate VEGF gene expression via heme oxygenase-1 induction (67). Ozone has a remarkable oxidative capacity, and can be stabilized for topical use, and may offer a simpler and more feasible option (42).

## 5. Conclusion

This study verified the safety and efficacy of intradermal injections of high ozone concentrations with no adverse effects. In summary, this study showed that O3W intradermal injection could hasten acute skin wound repair in diabetic rats by stimulating collagen synthesis and fibroblast proliferation at the wound site and inducing growth factors such as VEGF and TGF. Therefore, O3W intradermal injection may be considered an alternative treatment strategy to improve cutaneous wound healing and could provide significant benefits to diabetic animals.

## Data availability statement

The original contributions presented in the study are included in the article/Supplementary material, further inquiries can be directed to the corresponding authors.

## Ethics statement

The animal study was approved by This experimental protocol was approved by the Mansoura University Animal Care and Use Committee with a documented code MU-ACUC (MV.R.22.11.25). The study was conducted in accordance with the local legislation and institutional requirements.

## Author contributions

MA: Conceptualization, Data curation, Formal analysis, Funding acquisition, Investigation, Methodology, Project administration, Resources, Software, Validation, Visualization, Writing – original draft, Writing – review & editing. AH: Conceptualization, Data curation, Formal analysis, Funding acquisition, Methodology, Project administration, Software, Supervision, Visualization, Writing – original draft, Writing – review & editing. HA: Methodology, Project administration, Resources, Supervision, Visualization, Writing – review & editing, Data curation, Funding acquisition. RF: Conceptualization, Data curation, Formal analysis, Project administration, Resources, Software, Supervision, Validation, Writing – review & editing, Funding acquisition, Methodology. MR: Conceptualization, Data curation, Formal analysis, Investigation, Project administration, Software, Supervision, Writing – review & editing, Resources, Validation, Visualization. AAA: Conceptualization, Data curation, Investigation, Methodology, Software, Supervision, Writing – review & editing, Formal analysis, Project administration. WM: Conceptualization, Data curation, Investigation, Methodology, Software, Supervision, Validation, Writing – review & editing. IR: Conceptualization, Formal analysis, Funding acquisition, Investigation, Methodology, Project administration, Resources, Software, Supervision, Validation, Writing – original draft, Data curation, Visualization, Writing – review & editing. FZ: Conceptualization, Funding acquisition, Investigation, Project administration, Resources, Software, Supervision, Formal analysis, Methodology, Validation, Writing – original draft. SO: Conceptualization, Data curation, Funding acquisition, Project administration, Resources, Software, Supervision, Writing – review & editing, Investigation. AFA: Conceptualization, Data curation, Formal analysis, Investigation, Methodology, Project administration, Software, Supervision, Validation, Writing – review & editing. AR: Conceptualization, Data curation, Funding acquisition, Investigation, Methodology, Resources, Software, Supervision, Writing – review & editing. AE: ____.
