# Supplementary material for: Ozonated saline intradermal injection: promising therapy for accelerated cutaneous wound healing in diabetic rats
Source: Front Vet Sci. 2023 Nov 6;10:1283679. doi: 10.3389/fvets.2023.1283679 (PMC10657902; doi:10.3389/fvets.2023.1283679)
Supplement: Supplementary file 1 [file Data_Sheet_1.pdf]

Supplementary Material

1 Supplementary Figures

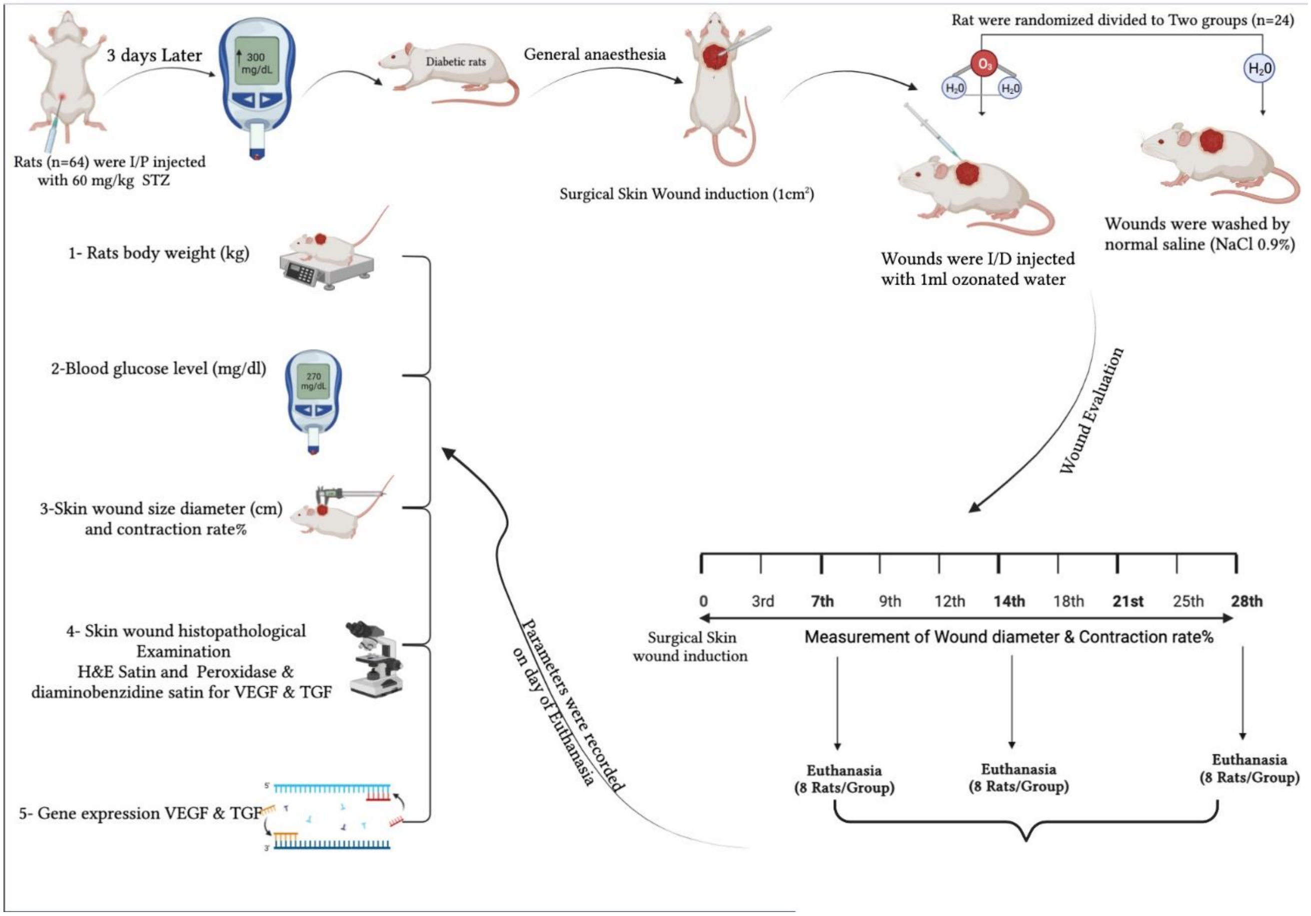

Supplementary Figure 1. The schematic cartoon of the experimental strategy. It was designed by the authors of the manuscript (created with Biorender.com with permission).

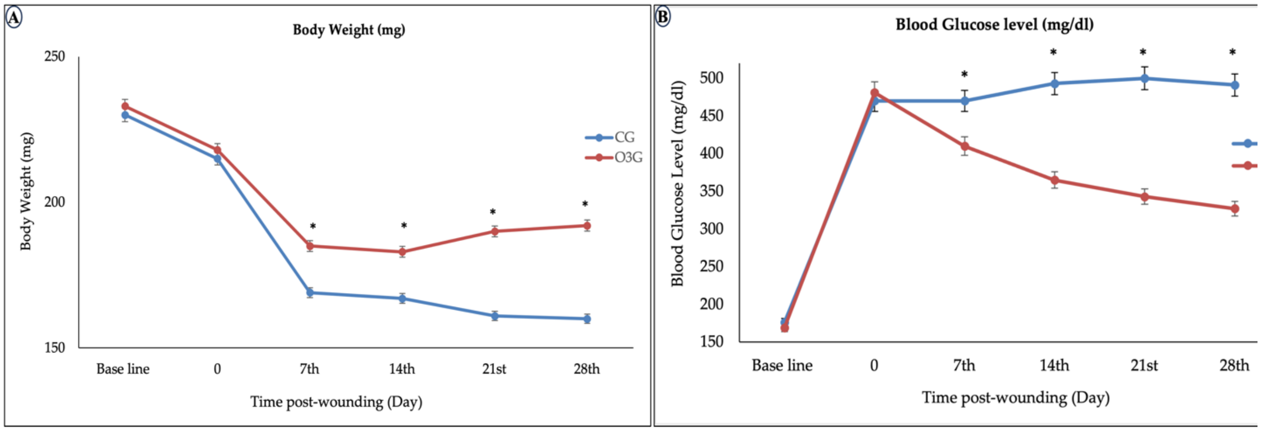

Supplementary Figure 2. Level of **(a)** body weight (mg) and (**b)** blood glucose level (mg/dl) in rats before induction of diabetes mellitus in rats and after diabetic induction at 0, 7th, 14th, 21st, 28th day post- wounding (mean ± SD).

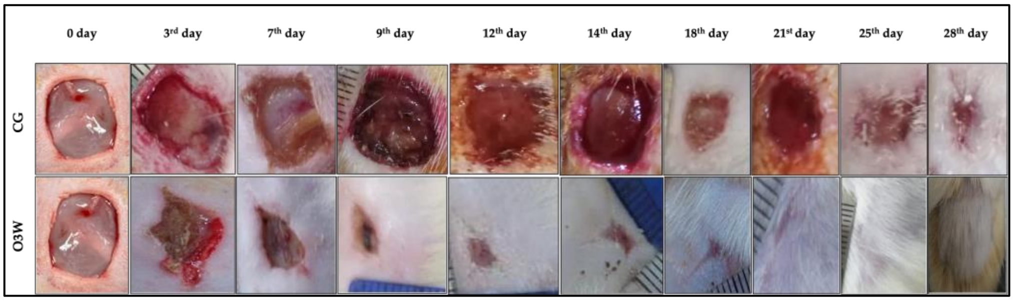

Supplementary Figure 3. Photographs of wound size in a control (CG) and an ozonated water (O3W) group during skin wound healing in rats at 0, 3rd, 6th, 9th, 12th,15th, 21st, 25th, and 28th days postoperatively.

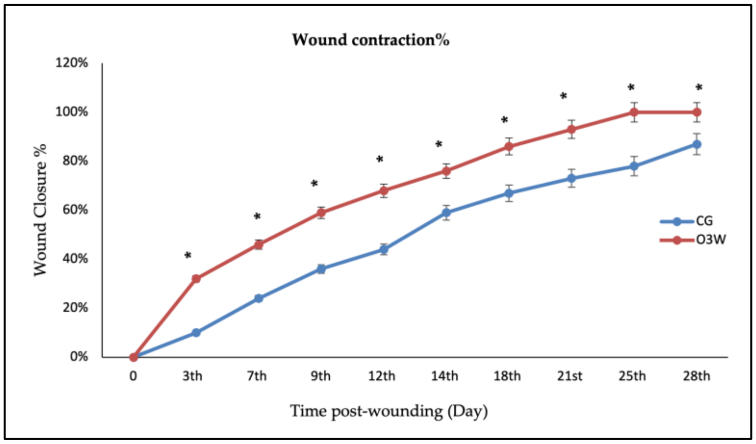

Supplementary Figure 4. The wound closure rate % in the control (CG) and ozonated water (O3W) groups during skin wound healing in rats at 0, 3rd, 6th, 9th, 12th,15th, 21st, 25th, and 28th days post-wound induction.

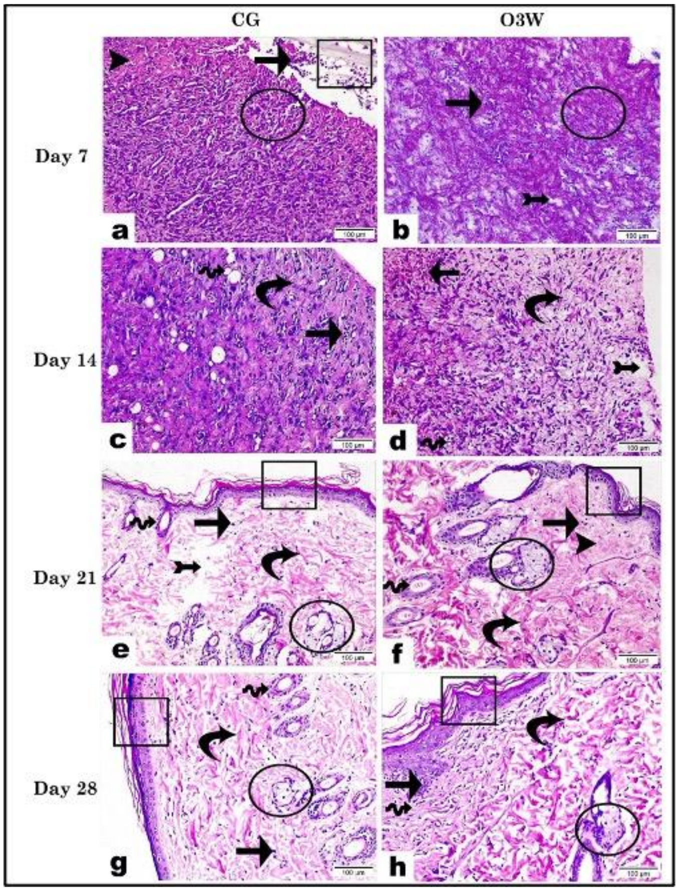

Supplementary Figure 5. Photomicrographs demonstrated the histopathological alterations in skin wound tissue sections between a control (CG) and an ozonated water (O3W) group (Hematoxylin & Eosin Stain, x200 & Scale Bar= 100μm) at the 7th, 14th, 21st, and 28th days post-wounding. Infiltration of mononuclear inflammatory cells (Arrow); granulation tissue (Arrowhead); free RBCs, fibrin threads, and epidermal layer (rectangle); bands of collagen fibers (circle); newly formed blood vessels (wave arrow); thick bands of collagen (curvy arrow); interstitial edema (arrow with tail); newly formed skin glands (circle).

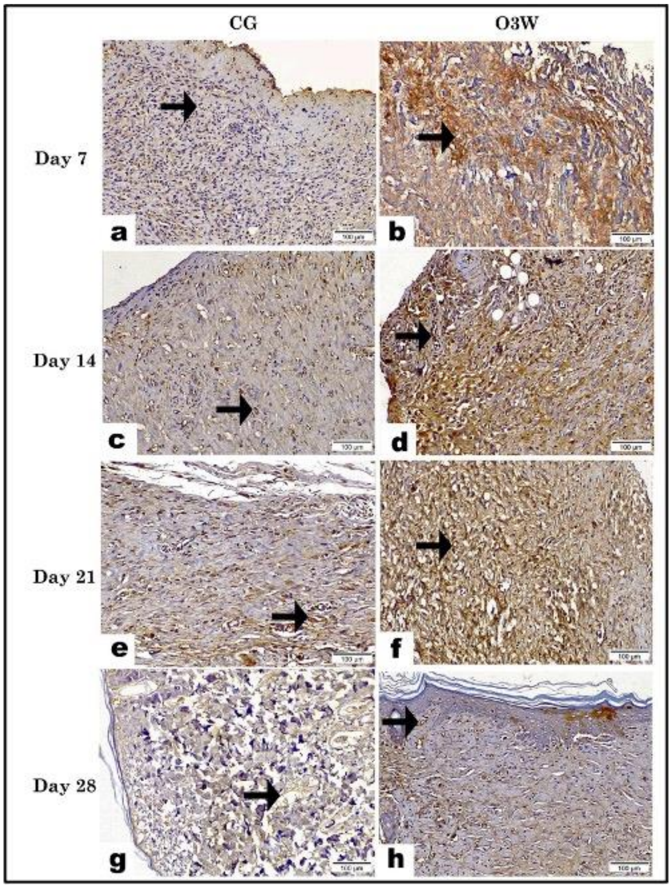

Supplementary Figure 6. Photomicrographs displayed the reactivity of VEGF in skin tissue sections between a control (CG) and an ozonated water (O3W) group (VEGF Antibody, Magnification Power= x200 & Scale Bar= 100µm) at days 7th, 14th, 21st and 28th post-wounding. Arrows are indicated by blood vessels.

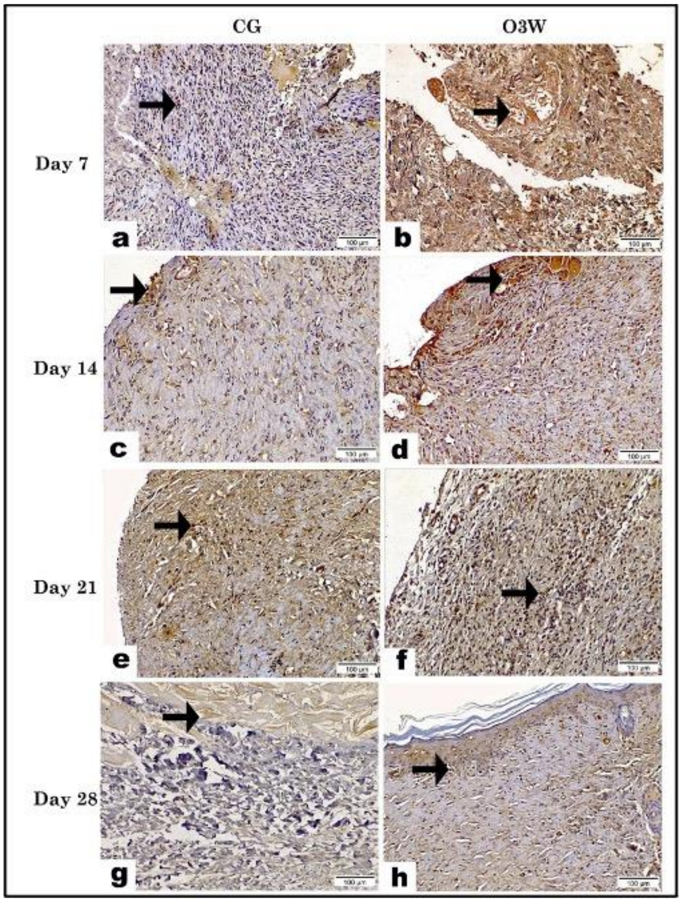

Supplementary Figure 7. Photomicrographs presented the expression of TGFβ in skin tissue sections between inspected groups (TGFβ Antibody, Magnification Power= x200 & Scale Bar= 100μm) on days 7th, 14th, 21st, and 28th post-wounding. Arrows indicate TGF*β* nuclear reactivity.

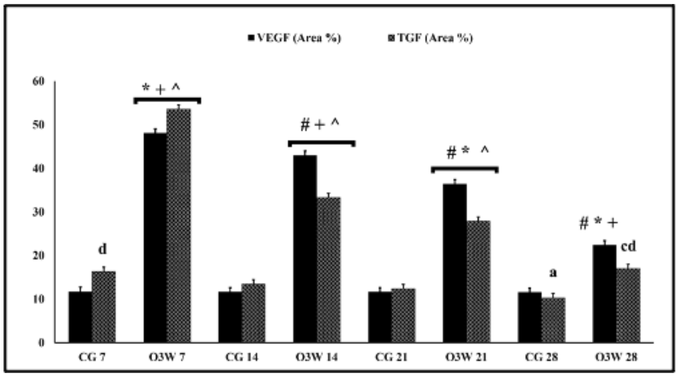

Supplementary Figure 8. The immune scoring area % of VEGF and TGFβ in skin tissue sections between a CG and an O3W group at 7th, 14th, 21st, and 28th post-wounding. Values expressed as Mean ± SD (area %), # significant vs. negative O3W 7, * significant vs. O3W 14, + significant vs. O3W 21, ^ significant VS O3W 28. a significant vs. CG 7, c significant vs. CG 21, d significant vs. CG 28. Different superscript (+, #, ^, *, a, c, d) indicate statistically significant differences at $P \leq 0.001$.

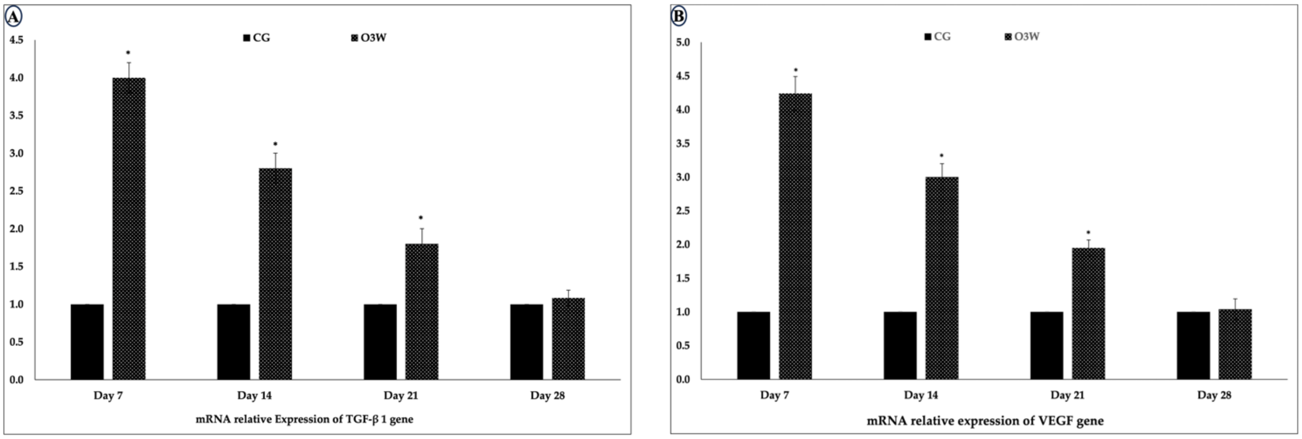

Supplementary Figure 9. Mean and standard deviation of relative expression affinity genes between control and O3W groups at 7th, 14th, 21st, and 28th days post-wound induction in diabetic rats: (a) VEGF mRNA gene (b) TGFβ1 mRNA gene.
